# Improved Optical Waveguide Microcantilever for Integrated Nanomechanical Sensor

**DOI:** 10.3390/s19194346

**Published:** 2019-10-08

**Authors:** Yachao Jing, Guofang Fan, Rongwei Wang, Zeping Zhang, Xiaoyu Cai, Jiasi Wei, Xin Chen, Hongyu Li, Yuan Li

**Affiliations:** 1 Key Laboratory of All Optical Network and Advanced Telecommunication Network, Ministry of Education, Institute of Lightwave Technology, Beijing Jiaotong University, Beijing 100044, China; 18120074@bjtu.edu.cn (Y.J.); 18120020@bjtu.edu.cn (R.W.); 18125090@bjtu.edu.cn (Z.Z.); 2 Shanghai Institute of Measurement and Testing Technology, National Center of Measurement and Testing for East China, National Center of Testing Technology, Shanghai 201203, China; caixiaoyu@simt.com.cn (X.C.); weijs@simt.com.cn (J.W.); 3 Department of Instrument Science and Engineering, Shanghai Jiao Tong University, Shanghai 200240, China; xchen.ie@sjtu.edu.cn; 4 College of Mechanical and Electronic Engineering, Shandong University of Science and Technology, Qingdao 266590, China; lihy08@gmail.com

**Keywords:** optical waveguide cantilever sensor, buffer, sensitivity

## Abstract

This paper reports on an improved optical waveguide microcantilever sensor with high sensitivity. To improve the sensitivity, a buffer was introduced into the connection of the input waveguide and optical waveguide cantilever by extending the input waveguide to reduce the coupling loss of the junction. The buffer-associated optical losses were examined for different cantilever thicknesses. The optimum length of the buffer was found to be 0.97 μm for a cantilever thickness of 300 nm. With this configuration, the optical loss was reduced to about 40%, and the maximum sensitivity was more than twice that of the conventional structure.

## 1. Introduction

In recent years, the development of micro processing and manufacturing technology has accelerated the research progress of nanomechanical sensors [1,2,3,4,5]. Nanomechanical sensors have been widely studied in biological, chemical, and environmental protection sensor applications because of their high sensitivity and capacity for integration [6,7,8]. The sensitivity of nanomechanical sensors is mainly affected by the readout method, relying on optical sensors used in atomic force microscopy or interference methods [9]. An alternative method has been developed that uses an optical waveguide cantilever (OWC) to detect the deflection or resonance change of cantilevers [10,11,12,13]. In this method, the light coupled into the optical waveguide cantilever and emitted from the free-end of the cantilever travels across a small gap and is captured by an output waveguide (OW). The major benefit of this method is the higher integration of cantilever arrays that can provide highly sensitive readouts. There are many reports about optical waveguide cantilevers based on different material platforms, for example, SiO_2_ [10], polymer [14], InP [15], and Si [16] as the cantilever waveguide.

In this study, we examined material platforms using Si_3_N_4_ as the optical waveguide and SiO_2_ as the cantilever to explore an optical waveguide cantilever system. An improved optical waveguide cantilever system was developed to improve optical sensitivity. Unlike the conventional structure, the proposed structure extends the input waveguide (IW) on the optical waveguide cantilever to reduce the coupling loss of the input waveguide and the cantilever. The finite element method was used to evaluate the proposed structure and for comparison with the conventional structure.

## 2. Improved Optical Waveguide Cantilever Sensor

**Figure 1:**
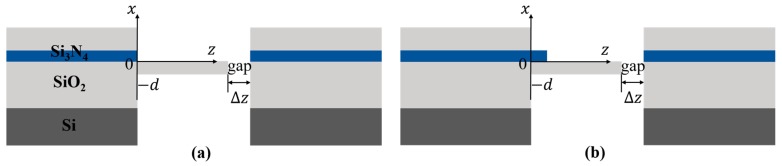
Schematic of the optical waveguide sensors: (**a**) conventional structure and (**b**) improved structure.

A conventional structure has been presented by several groups [10,11,12,13], as shown in Figure 1a. The structure of the sensor includes an IW, an OWC, and an OW. The light from a laser after propagating through the input waveguide is coupled into the microcantilever mainly by the evanescent field of the electromagnetic wave. Then, light exiting the microcantilever free-end propagates across the gap and is coupled into the output waveguide. The principle of operation is based on the dependence of the coupling efficiency between the cantilever and output waveguide considering their misalignment with respect to each other. When the position of the microcantilever free-end changes, the light coupled into the output waveguide also changes.

In the conventional structure (Figure 1a), the coupling loss of the input waveguide and the cantilever is larger due to the abrupt step on the junction, which affects the output power at the free-end of the cantilever and leads to poor optical sensitivity. Normally, a taper should be introduced to change the size and shape of the optical mode to achieve high coupling efficiency for the connection. To do this, the taper must operate adiabatically by increasing or decreasing the size of the taper cross section very slowly; that is, the local first-order mode of the waveguide should propagate through the taper while undergoing relatively little mode conversion to higher-order or radiation modes [17,18,19,20].

However, the taper in our structure requires complex fabrication technology. To improve the optical sensitivity and make the fabrication process as simple as possible, a buffer on the connection of the input waveguide and cantilever was introduced as a substitution for the adiabatic taper (Figure 1b) to reduce the coupling loss.

According to the principle of optical waveguide microcantilever sensors, the cantilever displacements of bending in the *x* direction lead to a change of the coupling efficiency between the cantilever and the output waveguide. Hence, one can know the bending displacement by monitoring the coupling efficiency. The coupling efficiency can be calculated using the overlap integral [21]:

(1) $$\Gamma \left(x,\Delta z\right)=\frac{{\left(\int_{-\infty }^{\infty }E_{gap}\left(x,\Delta z\right)E_{y}^{*}\left(x\right)dx\right)}^{2}}{\int_{-\infty }^{\infty }E_{gap}\left(x,\Delta z\right)E_{gap}^{*}\left(x,\Delta z\right)dx\int_{-\infty }^{\infty }E_{y}\left(x\right)E_{y}^{*}\left(x\right)dx},$$

where $E_{gap}\left(x,\Delta z\right)$ is the electric field distribution of the light exiting the cantilever at the distance of Δz, and $E_{y}\left(x\right)$ is the distribution of the electric field of the output waveguide.

In this study, we only considered the vertical direction of the cantilever and the input and output waveguides for operation in the transverse electric (TE) mode. Thus, the transverse function $E_{y}\left(x\right)$ has a general form from Maxwell’s wave equation considering a basic three-layer waveguide structure model [21]:

(2) $$E_{y}=\{\begin{array}{c} Ae^{-\gamma_{s}x}\;x>0\\ A\left(\cos\left(k_{x}x\right)-\frac{\gamma_{c}}{k_{x}}\sin\left(k_{x}x\right)\right)\;-d<x<0\\ A\left(\cos\left(k_{x}d\right)+\frac{\gamma_{c}}{k_{x}}\sin\left(k_{x}d\right)\right)e^{r_{s}\left(x+d\right)}\;x<-d \end{array},$$

where $k_{x}=k_{0}\sqrt{n_{f}^{2}-N^{2}}$, $\gamma_{c}=k_{0}\sqrt{N^{2}-n_{c}^{2}}$, and $\gamma_{s}=k_{0}\sqrt{N^{2}-n_{s}^{2}}$ are the transverse propagation constants of the waveguide, cladding, and substrate, respectively; $k_{0}=\frac{2\pi }{\lambda }$ is the wave in vacuum with a wave length of *λ*; *d* is the thickness of the core; *N* is the effective refractive index, which can be obtained by Equation (3); $n_{c}$, $n_{f}$, and $n_{s}$ are the refractive indexes of the cladding, core, and substrate, respectively; and *A* is the amplitude of the electric field.

Light propagates across the waveguide in the form of a guided mode. Different guided modes correspond to different effective refractive indexes. For asymmetric planar waveguides in TE modes, the corresponding eigenvalue equation can be obtained by applying the boundary conditions and the electric field continuity condition:

(3) $$V\sqrt{1-b}=m\pi +\tan^{-1}\sqrt{\frac{b}{1-b}}+\tan^{-1}\sqrt{\frac{b+a}{1-b}},$$

where $b=\frac{N^{2}-n_{s}^{2}}{n_{f}^{2}-n_{s}^{2}}$ is the normalized waveguide refractive index, V is the normalized waveguide thickness, and $a=\frac{n_{s}^{2}-n_{c}^{2}}{n_{f}^{2}-n_{s}^{2}}$ is the asymmetric part of the waveguide.

The optical sensitivity is defined as the derivative of the coupling efficiency and the free-end bending displacement of the cantilever beam:

(4) $$\mathrm{Sens}=\frac{\partial \Gamma }{\partial x}$$

## 3. Results and Discussion

Finite element analysis was performed to evaluate the proposed structure. In the simulation, the input and output waveguide was 80 nm thick and made of silicon nitrous oxide ($n_{Si_{3}N_{4}}=2.0$), the optical waveguide cantilever was 90 μm long and made of silicon oxide ($n_{SiO_{2}}=1.46$), and the gap was 2 μm wide.

**Figure 2:**
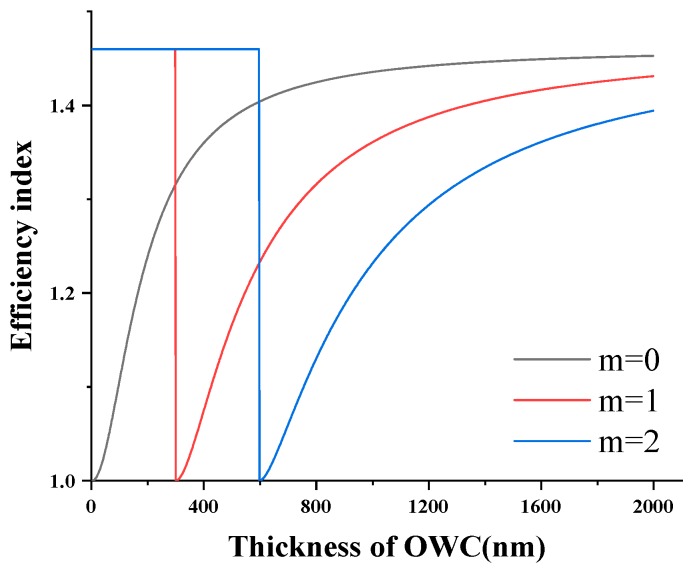
Effective index with the thickness of the optical cantilever.

In order to decide the thickness of the cantilever in the vertical direction, the guided modes of the cantilever waveguide were determined by the effective index method. The relationship of the effective index and the thickness of the cantilever are shown in Figure 2 for zero-, first-, and second-order modes. For operation at a higher sensitivity, the cantilever was in single mode in the vertical direction and was as thin as possible. However, the cantilever cannot be too thin, as it will not be able to support its own weight, which will lead to low coupling efficiency with the fiber [22]. Based on the index shown in Figure 2, the thickness of the cantilever was chosen as 300 nm.

**Figure 3:**
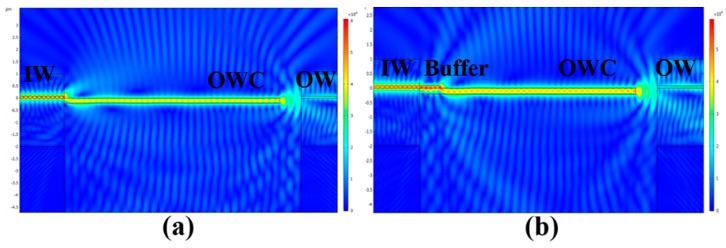
Electric field distribution for the input waveguide and cantilever of (**a**) the conventional structure and (**b**) the improved structure with a buffer.

In the proposed structure, a buffer was introduced into the connection of the input waveguide and the cantilever to reduce the coupling loss due to the abrupt step. The electric field distribution is shown in Figure 3 for the conventional and improved structures with a buffer. It can be seen from Figure 3a that when there was no buffer, the coupling mode changed from strong coupling to radiative modes. Comparing these two images shows that the buffer could significantly increase the coupling efficiency of the input waveguide and cantilever.

**Figure 4:**
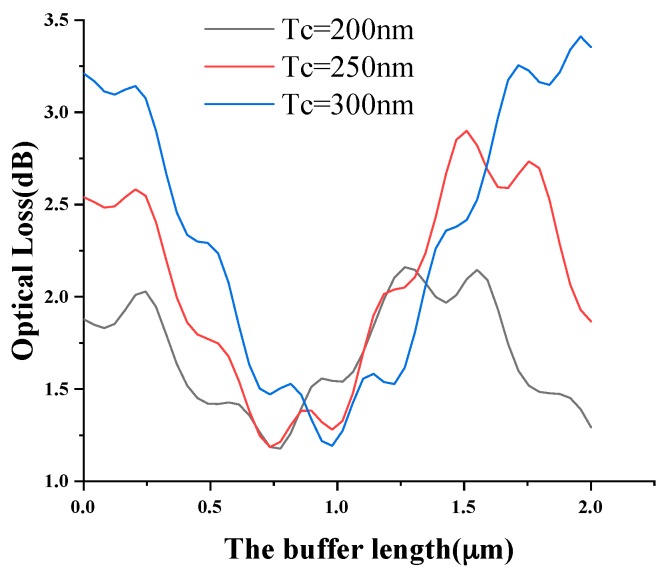
The optical loss in the air with the buffer length for different OWC thicknesses.

To evaluate the buffer, the optical losses on the free-end of the cantilever were calculated as a function of the buffer length for thicknesses of 200, 250, and 300 nm, as shown in Figure 4. The optical losses were mainly from the coupling losses of the input waveguide and the cantilever, and excessive loss of optical power may have reduced the sensitivity. As shown in Figure 4, the curves were similar to the sine shape of the curve, and the optimal length of the buffer was different for different cantilever thicknesses. A 0.97 μm length buffer was chosen for the thickness of 300 nm.

**Figure 5:**
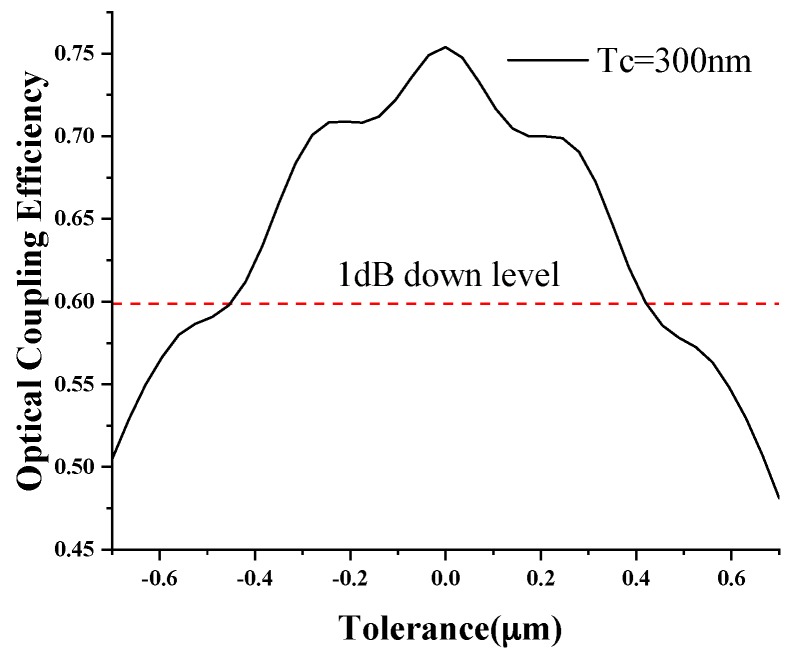
The optical coupling efficiency with the fabrication tolerances of the 0.97 um length buffer.

In the manufacturing process, it is almost impossible to achieve a completely accurate device due to technological limitations and other factors, and it is crucial to ensure that acceptable detection errors caused by the device size difference are within a certain range. Fabrication tolerances with the 0.97 μm length buffer were analyzed (Figure 5). Within the tolerance range −0.4 to 0.4 μm, the optical coupling efficiency decreased by less than 1 dB, which was enough to ensure efficiency that was greater than without the buffer and that the device would not produce large deviations due to the size difference.

Sensitivity depends on the change of the optical power coupled into the output waveguide during the deflection of the cantilever. In order to model a desired cantilever bending process, solid mechanics was used to simulate cantilever bending under stress. The gap was 2 μm, the length of the buffer was 0.97 μm, and the thickness of the cantilever was 300 nm.

**Figure 6:**
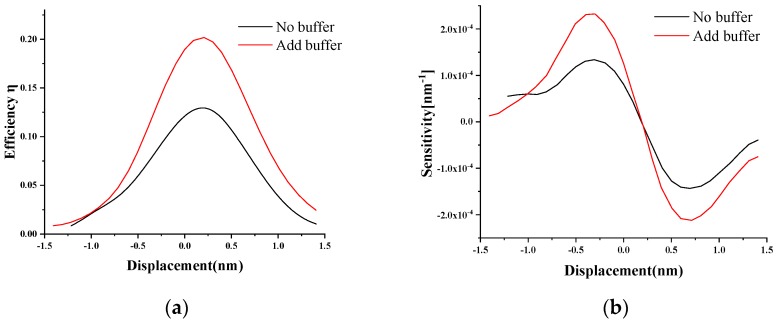
(**a**) The coupling efficiency. (**b**) The sensitivity with the displacement of the cantilever for the conventional structure and the improved structure with a buffer.

In this study, the change of the coupling efficiency between the OWC and the OW was simulated when the force at the end of the OWC deflected, and the change of device sensitivity with the displacement was obtained using the derivative of coupling efficiency and free-end deflection according to Equation (4). The optical efficiency for different cantilever bendings was calculated, as shown in Figure 6a. Using the derivative of the curve, the sensitivity was obtained (Figure 6b). As shown in Figure 6, the optical efficiency and sensitivity of the structure with a buffer had obvious improvements compared with the conventional structure. The sensitivity of the structure with a buffer was more than twice as high as the conventional structure. Additionally, the most dramatic change of optical power occurred when the cantilever bent slightly. The position of maximum sensitivity was not at the place when the OWC was completely aligned with the IW, but rather the position where the cantilever beam was slightly deflected.

## 4. Conclusions

In this paper, an improved optical microcantilever waveguide sensor was proposed. Unlike the conventional structure, a buffer was introduced into the connection of the input waveguide and the cantilever by extending the input waveguide, which reduced the coupling loss of the input waveguide and the cantilever. The buffer length was studied for its capacity to reduce optical loss for different cantilever thicknesses. A 300 nm thick cantilever beam and a 0.97 μm length buffer were selected to improve the coupling efficiency of the input waveguide and the cantilever. In order to ensure the stability of the device, the dimensional tolerances of the buffer in the manufacturing process were also analyzed. The performances of conventional and improved cantilever sensors were compared, and the coupling efficiency and the sensitivity were discussed as the key parameters of the device. The results showed that the coupling efficiency and the sensitivity had obviously improved.
